# Party capacity, state capacity, and pandemic governance: a fuzzy-set qualitative comparative analysis of COVID-19 prevention and control outcomes in 52 African countries during the early stage

**DOI:** 10.3389/fpubh.2026.1947309

**Published:** 2026-10-08

**Authors:** Song Li, Zhipeng Wang

**Affiliations:** School of Marxism, East China University of Political Science and Law, Shanghai, China

**Keywords:** African politics, COVID-19, pandemic governance, qualitative comparative analysis, ruling party capacity, state capacity

## Abstract

**Introduction:**

During the early phase of the COVID-19 pandemic in Africa, from March to May 2020, significant variations emerged among countries regarding the implementation of containment policies. During this period, viewed strictly from a public health perspective, social distancing and lockdowns were considered critical for curbing transmission. However, epidemic control outcomes varied considerably across countries. This variation raises questions about the independent and interactive influence of socioeconomic conditions on these outcomes, and about how political factors, including state capacity and party politics, moderate or mediate these influences, especially in resource-constrained contexts.

**Methods:**

This article employs fuzzy-set Qualitative Comparative Analysis (fsQCA) with 52 African countries as case studies to investigate the combination of conditions for COVID-19 containment success.

**Results:**

The analysis reveals three equifinal configurations: a party-led high-risk response pathway, an administrative compensation pathway, and a coercive compensation pathway. Party capacity is not a generally applicable determinant; its role is contingent upon the specific governance context. Under the structural constraint of healthcare deficiency, distinct dimensions of state capacity operate through differentiated compensatory mechanisms.

**Discussion:**

The paper makes two theoretical contributions: conceptualizing party capacity as a crisis-contingent rather than a generalized governance asset, and disaggregating state capacity into administrative and coercive dimensions with distinct compensatory functions. It also makes a methodological contribution by adopting a configurational approach that examines combinations of conditions rather than the net effects of individual factors.

## 1 Introduction

In early 2020, Africa faced significant risks from the COVID-19 pandemic, yet the outcomes were less severe than initially anticipated. Governments across the continent implemented a range of interventions, including social distancing measures, lockdowns, and travel restrictions, although the degree of enforcement varied considerably. A study of 22 African countries found that school closures and bans on public gatherings were universally adopted. However, substantial differences existed in stay-at-home orders, policy stringency, exemptions for healthcare services, and law enforcement practices (1). This variation created a natural policy laboratory for comparative analysis.

Against this backdrop, this study examines how combinations of party capacity (PC), state capacity, and social structural factors are configurationally associated with COVID-19 containment during the early phase of the “Great Lockdown” in African countries. Specifically, it asks three questions: (1) which configurations of ruling-party capacity, state capacity, and governance context are sufficient for high early-stage pandemic governance effectiveness; (2) whether party capacity operates as a generally applicable governance resource or as a context-dependent one; and (3) where healthcare capacity is weak, which dimension of state capacity—administrative or coercive—compensates for it, and through what mechanism. Through a comparative multi-case analysis of African countries, this study seeks to explain the variation in COVID-19 containment effectiveness across the continent.

The findings of this study offer analytical insights rather than definitive causal claims. Theoretically, the study moves beyond debates concerning the effectiveness of individual policy measures and instead examines how interactions among PC, state capacity, and societal conditions jointly shape policy trajectories and outcomes. It contributes to the literature on party politics and state capacity in developing countries by systematically incorporating socio-structural factors into the analytical framework of public health policy. All findings should be interpreted with caution given the exploratory nature of the QCA approach and the acknowledged limitations of reported mortality data.

## 2 Literature review

In 2020, African nations adopted robust initial countermeasures against the pandemic. Nevertheless, subsequent containment efficacy varied markedly across the continent. Experience gained from responding to major outbreaks may have influenced early COVID-19 responses in several African countries (2), alongside socioeconomic conditions, including demographic structures and spatial distribution. It is generally agreed that political factors play an important role in pandemic governance. Existing research explains African pandemic governance from three political perspectives.

These three perspectives are not mutually exclusive but progressively shift analytical focus from state institutions to societal actors and finally to the political organizations that mediate state-society relations. First, the state-centric approach prioritizes state capacity, a multidimensional construct pivotal to pandemic containment. In Mann's terms, state capacity refers to “infrastructural power,” that is, the ability to coordinate society and implement policy across national territory (3). Empirical evidence from Africa lends support: a PERC report indicates higher-than-expected institutional preparedness at the pandemic's onset (4), and governments with stronger state capacity responded more swiftly and coordinated more effectively (5). Cross-country analyses confirm that government effectiveness is significantly negatively correlated with COVID-19 mortality (6). However, critics caution that pandemic responses facilitated surveillance expansion under the guise of public health, raising concerns about civil liberties and democratic accountability. Emergency powers tend to persist beyond crises, and enforcement infrastructure may be repurposed for non-health objectives (7). These concerns underscore the need to examine not only state effectiveness but also its legitimacy and institutional checks (8). In sum, while the state-centric approach offers a compelling framework, it must be complemented by attention to the political and social contexts within which state capacity operates.

Second, the society-centric approach emphasizes non-state actors. The society-centric perspective in African pandemic studies focuses on non-state actors, emphasizing the influence of ethnic politics and pluralistic political structures on governance (9). The pandemic has exacerbated government trust deficits in Africa, with citizens increasingly distrusting health experts and government officials; in this credibility vacuum, civil society organizations have emerged as trusted alternative actors (10, 11). The Ebola crisis significantly eroded public trust in governments (12). In this context, locally trusted actors, particularly faith leaders, civil society organizations, and community volunteers, played a critical role in the epidemic response (12, 13). Critics, however, argue that early society-centric perspectives tended to idealize civil society or rely on reductive “neo-patrimonialism” frameworks, thereby overlooking the institutional diversity of African arrangements. Although civil society organizations have filled critical gaps in service provision and advocacy, grounded in local trust and traditional systems, deep-seated obstacles persist, including ethnic cleavages, distrust of state institutions, and the politicization of crises. This perspective offers important insights into state-society relations, but should be complemented by other analytical frameworks to avoid one-sided interpretations.

Third, the party-centric perspective highlights that robust political parties have played a pivotal role in Africa's state-building trajectory. From a party-centric perspective, scholars examine parties' power operations and decision logics in crisis governance. Tanzania shows how the ruling party used “economy-first” rhetoric to suppress expert authority. Party system variations shape responsibility allocation (14). Nigeria's federal government avoids risks, leaving subnational politicians to bear costs, while South Africa centralizes responsibility, rooted in party affiliation and electoral incentives. A strong party or executive core enables swift decisions, but power concentration raises democratic and human rights concerns (8, 15). Overall, pandemic response in Africa should be understood not as a purely technical exercise, but as a form of power practice embedded in the competitive and organizational logic of party politics (16).

Reviewing state-, society-, and party-centric perspectives shows each has merits and limits. The state approach ignores interaction and compensation among capacity dimensions under healthcare deficits. The society approach idealizes civil society without specifying substitution conditions. The party approach treats capacity as generalized, missing context dependence. We therefore ask: what configurations of party capacity, state dimensions, and context produce high pandemic effectiveness? Is party capacity universal or context-specific? When healthcare is weak, which state dimension (administrative or coercive) compensates, and how?

### 2.1 Analytical framework and the conditions

This study employs a fuzzy-set qualitative comparative analysis (fsQCA) approach to examine the configurational conditions that lead to different COVID-19 control outcomes across African countries.

#### 2.1.1 Governance context of pandemic control in Africa

Governance context conditions constitute foundational constraints on pandemic control. These include demographic structure, such as age composition. The median age in Africa is only 19.7 years, which reduces population-level risk of severe cases and mortality (17). Additionally, social capital and local experience play crucial roles. Social capital has been shown to facilitate epidemic response, with higher levels of voluntary association participation correlating with shorter Ebola duration in West Africa (13).

#### 2.1.2 State capacity in African pandemic response: roles and limitations

State capacity is a multidimensional concept. This study conceptualizes it as governance capacity for policy development and implementation, while also analyzing the institutional, organizational, and political factors that shape it (18). Many African countries demonstrated rapid decision-making and implementation during the pandemic, which was pivotal to their initial containment success. For example, a UNECA report notes that 38 countries in Africa closed their land borders and 17 closed their maritime borders as part of early COVID-19 responses (19). This “iron-fisted lockdown” approach even exceeded some states' routine governance modalities, reflecting a concentrated exercise of coercive state power in crisis situations. However, shaped by colonial legacies that disrupted indigenous governance and long neglected public health, African state capacity remains highly heterogeneous, leaving many states with the dual predicament of weak health infrastructure and severe resource shortages.

#### 2.1.3 Party capacity and political mobilization in pandemic response

PC emerges as a critical factor. First, the ruling party's stability and internal cohesion are foundational, ensuring both long-term governance capacity and consistent policy implementation. Second, a robust party organizational network and social mobilization capacity enable effective policy communication, public participation, and allocation of scarce resources. Third, a nationwide party network is decisive in establishing a unified control system across the entire country. Conversely, intense political polarization and low inter-party cooperation have undermined pandemic prevention efforts, resulting in weak implementation and poor outcomes.

Given the complexity of pandemic governance, this framework goes beyond approaches that rely solely on regime-type comparisons or isolated measures of state capacity. It requires an integrated approach that considers formal political institutions alongside informal governance contexts and dynamic inter-and intra-party-political processes.

## 3 Research design and data processing

### 3.1 Unit of analysis and case selection

This study examines 52 African member states of the United Nations. Eswatini (SWZ) and South Sudan (SSD) were excluded because of missing data for key variables and concerns regarding data comparability. The final sample includes 52 countries from North, East, West, Central, and Southern Africa. These countries exhibit substantial variation in geographical location, political regime type, economic development, and governance performance, thereby providing a diverse set of cases for configurational analysis using QCA (20–23).

The temporal cross-section is set at May 31, 2020, corresponding to the early phase of the first wave of the COVID-19 pandemic in Africa. This time point was selected for three reasons. First, by this date, most countries had already implemented major interventions, including border controls, social distancing measures, school closures, and workplace restrictions, making initial governance outcomes observable. Second, vaccination campaigns had not yet commenced, thereby eliminating potential confounding effects arising from cross-national differences in vaccine access and distribution. Third, governance outcomes during this period primarily reflected the implementation of non-pharmaceutical interventions (NPIs), which provide an appropriate basis for assessing party and state capacities (24).

### 3.2 Outcome variable

This study operationalizes early-stage governance effectiveness as “low mortality rates” (Y), measured using COVID-19 deaths per million population (Deaths_per_million) as of May 31, 2020. COVID-19 data were obtained from the WHO COVID-19 Dashboard (25), while population data were sourced from the World Bank database (26).

The distribution of the original mortality variable is highly right-skewed (skewness = 4.05), with several countries reporting either zero or extremely low mortality rates. Direct fuzzy-set calibration of the raw data could therefore allow extreme values to exert disproportionate influence on membership scores. To address this issue, the study first applies a logarithmic transformation, log (Deaths_per_million + 1), and subsequently employs Ragin's direct calibration method. The 5th, 50th, and 95th percentiles of the transformed variable are used as the thresholds for full exclusion, the crossover point, and full inclusion, respectively (20) to calibrate membership in the “high mortality rate” set. The complement of this set is then used to construct the “low mortality rate” set (*Y* = 1 − BAD_deaths_per_million), in which higher membership scores indicate more effective pandemic governance outcomes.

It should be noted that COVID-19 statistics in African countries were influenced by substantial cross-national variation in testing capacity and reporting systems (27). Previous research suggests that official figures considerably underestimated actual infection and mortality levels across the continent, with the ratio of actual to confirmed infections potentially reaching 100:1 (28). Nevertheless, population-based mortality indicators are generally less directly affected by differences in testing capacity and strategies than confirmed case counts (29, 30). Accordingly, population-standardized COVID-19 mortality, including deaths per million population, has been employed as an outcome measure in comparative studies of early pandemic performance, including research specifically focused on African countries (30, 31). Certainly, cross-national differences in epidemic timing, mortality registration, demographic structure, and other epidemiological factors may still affect observed mortality. Accordingly, Y, defined as membership in the set of countries with low reported population-standardized COVID-19 mortality, should not be interpreted as a direct measure of governance effectiveness. Rather, within the temporal and comparative scope of this study, it is used as a contextually bounded but acceptable proxy for early-stage pandemic governance performance.

### 3.3 Condition variables

This study selects five condition variables representing the dimensions of PC, state capacity, and governance risk. Table 1 summarizes the operationalization, measurement, and calibration procedures for each condition variable.

**Table 1:** Variable definition and operationalization scheme.

Category	Label	Indicator(s)	Calibration method
Outcome	*Y*	COVID-19 deaths per million(log)	Direct calibration; *Y* = 1 − BAD
Party capacity	PC	v2palocoff; v2paactcom; v2pasoctie	Each calibrated separately (anchors −1/0/+1); intersection of three
State capacity (admin)	AE	GE_2019; CC_2019	Each calibrated separately; intersection of two
State capacity (coercive)	TC	v2svstterr 2019	Direct calibration
State capacity (health)	HC	Doctors_per_10k 2019(log)	Direct calibration
Governance context	PR	International tourism arrivals 2019(log); Population living in slums 2018	Each calibrated separately; intersection of two

PC is the core explanatory variable, characterizing the ruling party's^1^ organizational mobilization, resource integration, and social embeddedness in crisis situations. Huntington identifies four dimensions of political institutionalization, adaptability, complexity, autonomy, and coherence, which provide a theoretical reference for defining PC (32). Additionally, Randall and Svåsand propose an analytical framework of party institutionalization that includes “systemness,” “value infusion,” “decisional autonomy,” and “reification,” offering a more operationalizable pathway for empirical measurement (33). Of these dimensions, the present study operationalizes those concerned with organizational infrastructure: Huntington's complexity and Randall and Svåsand's systemness and reification. These are the attributes through which a party's societal presence is converted into implementation capacity under crisis conditions. Coherence and value infusion concern internal discipline and normative attachment, and decisional autonomy concerns the party's independence from external sponsors; all three are conceptually distinct from reach and are not aggregated into the condition set. Restricting the construct in this way narrows its scope but sharpens its interpretability, and avoids the composite-index problem whereby a single high-scoring dimension can offset structural weakness elsewhere.

The V-Party index captures ruling parties' organizational infrastructure, but this does not automatically translate into governance effectiveness. In many African states, party networks operate within neo-patrimonial systems, serving patronage distribution and electoral mobilization in routine political times. A high V-Party score may therefore reflect extensive clientelistic reach rather than governance-oriented capacity. However, Crises, particularly public health emergencies, can repurpose these networks for governance functions. Three crisis features enable this conversion: the delegitimization of routine patronage practices, strong state incentives for mass mobilization, and heightened public receptivity to authoritative guidance. Thus, organizational capacity constitutes a necessary foundation, but crisis serves as the catalyst that activates and redirects this capacity toward governance effectiveness. This study examines precisely this conversion mechanism under COVID-19 conditions.

This paper integrates three indicators from the V-Party dataset: local party organization coverage (v2palocoff), local party organization strength (v2paactcom), and ties with affiliated social organizations (v2pasoctie) (34) Direct calibration uses theoretical anchors of −1, 0, and +1^2^; their intersection (minimum) is then taken. Aggregation by intersection imposes a stricter measurement standard for party organizational capacity than a composite average would, because a ruling party qualifies as organizationally strong only where all three dimensions are simultaneously present. This requirement follows from their non-compensatory relationship, as local presence without sustained activity, or active local units without linkage to organized social constituencies, cannot be converted into implementation capacity during a health emergency.

State capacity is generally understood as multidimensional, encompassing the capabilities required to formulate and implement collective decisions across state functions (35). Given the specific demands of pandemic governance, this study operationalizes these capabilities through three functionally relevant dimensions: administrative execution, territorial control, and healthcare capacity. First, administrative execution capacity (AE) combines Government Effectiveness (GE_2019) and Control of Corruption (CC_2019) from the World Bank's Worldwide Governance Indicators (36). Each indicator is separately calibrated using Ragin's direct method with the 5th, 50th, and 95th percentiles as anchors; ^3^their intersection (minimum) is then taken to reflect the “weakest-link” effect in policy implementation. Second, territorial control capacity (TC) from V-Dem measures the state's actual control over its territory (v2svstterr) (37). Full non-membership and full membership are anchored at the 5th and 95th percentiles of the sample distribution, while the crossover is set marginally above the sample median in order to avoid membership scores of exactly 0.50^4^. Third, healthcare capacity (HC) is measured by physicians per 10,000 population (Doctors_per_10k) from the WHO Global Health Observatory (38). After log transformation, log (Doctors_per_10k + 1), ^5^Ragin's direct calibration uses the 5th, 50th, and 95th percentiles as anchors.

Governance context-pandemic transmission risk (PR) is a variable capturing both international import risk and domestic spread risk. The former is proxied by 2019 inbound tourist arrivals (39), capturing pre-pandemic exposure to international mobility and hence the structural risk of imported infections (40). The measure is log-transformed, log (Tourism + 1), and then calibrated using Ragin's direct method with the 5th, 50th, and 95th percentiles as anchors.^6^ Domestic diffusion risk is measured by the share of the urban population living in informal settlements in 2018 (41), calibrated using the same percentile anchors. The informal-settlement share offers direct construct validity for domestic diffusion risk. Under the definition adopted for this series, a household is classified as living in an informal settlement where it lacks one or more of improved water, improved sanitation, sufficient living area, housing durability, and security of tenure. Two of these components, insufficient living area and inadequate or shared sanitation, directly shape exposure opportunities and infection control (42, 43), and are thus directly informative about the implementability of non-pharmaceutical interventions during the early stage of the pandemic. PR is obtained as the intersection (minimum) of the two calibrated scores. The raw data and calibrated set-membership scores for all 52 cases are provided in Supplementary Table 1.

### 3.4 Condition analysis

This paper first tests the necessity of each individual condition. QCA research typically adopts a consistency threshold of ≥0.90 as the criterion for a necessary condition and further assesses substantive explanatory significance using coverage and RoN (Relevance of Necessity) indicators (20, 22). No condition, in either its positive or its negated form, reaches the 0.90 consistency threshold. Table 2 reports the results of the necessity analysis for each individual condition.

**Table 2:** Single-condition necessity analysis results.

Condition	Consistency	Coverage	RoN
**PC**	0.766	0.641	0.609
**~PC**	0.389	0.581	0.810
**AE**	0.575	0.713	0.821
**~AE**	0.639	0.604	0.658
**TC**	0.702	0.706	0.749
**~TC**	0.520	0.597	0.739
**HC**	0.507	0.587	0.737
**~HC**	0.730	0.731	0.762
**PR**	0.415	0.881	0.961
**~PR**	0.788	0.566	0.438

“~” denotes the absence of a condition. Consistency and coverage are computed in fsQCA 4.1. RoN, calculated as Σ(1−*X*_*i*_)/Σ[1−*min*(*X*_*i*_, *Y*_*i*_)] following Schneider and Wagemann (22), is not reported by fsQCA 4.1 and was computed separately from the calibrated data. No condition reaches the 0.90 consistency threshold.

Prior to analyzing the sufficiency of condition configurations, it is necessary to construct a truth table that converts fuzzy-set scores into crisp sets for analysis. In truth table analysis, based on the dichotomous assignment of five condition variables (membership >0.5 indicates presence, < 0.5 indicates absence), a total of 2^5^ = 32 condition configurations are theoretically generated. Among these, 20 configurations are actually observed, covering all 52 cases, while the remaining 12 constitute logical remainders. In constructing the truth table, the frequency threshold is set to 1 to ensure that each included configuration contains at least one empirical case.

For outcome assignment, this paper adopts a dual screening criterion: raw consistency not lower than 0.80 and “Proportional Reduction in Inconsistency” (PRI) not lower than 0.65^7^ (22). Raw consistency identifies whether a configuration broadly constitutes a subset of the outcome set, while PRI excludes the “simultaneous subset” problem, where a configuration overlaps strongly with both the outcome set and its complement, rendering the direction of sufficiency ambiguous. According to this criterion, a total of 11 configurations were assigned a value of “1,” and the remaining 9 were assigned “0.” Table 3 presents the truth table for this study.

**Table 3:** Truth table.

PC	AE	TC	HC	PR	*N*	Raw consistency	PRI consistency	*Y*
0	0	1	0	1	1	0.980	0.945	1
0	1	0	0	1	1	0.980	0.942	1
1	1	0	0	1	1	0.968	0.896	1
1	0	0	0	1	1	0.966	0.888	1
1	0	1	1	1	1	0.940	0.828	1
0	1	1	0	0	1	0.935	0.846	1
1	0	0	1	1	1	0.931	0.742	1
0	1	0	0	0	2	0.904	0.724	1
1	1	1	0	0	3	0.895	0.799	1
1	1	0	0	0	2	0.873	0.682	1
0	0	0	1	1	1	0.864	0.584	0
0	1	0	1	1	1	0.822	0.531	0
1	0	1	0	0	4	0.818	0.657	1
1	0	0	1	0	3	0.770	0.397	0
0	0	0	0	0	2	0.756	0.431	0
1	0	0	0	0	8	0.748	0.455	0
0	0	0	1	0	4	0.745	0.437	0
1	0	1	1	0	4	0.716	0.459	0
1	1	1	1	0	5	0.691	0.459	0
0	1	1	1	0	6	0.661	0.411	0

A configuration is assigned “1” when raw consistency ≥ 0.80 and PRI ≥ 0.65; otherwise “0”. Two configurations (rows 11 and 12) meet the raw consistency criterion but fall below the PRI threshold and are therefore assigned “0”. The truth table is sorted by raw consistency in descending order.

Following the truth table construction, this paper derives three solutions (complex, parsimonious, and intermediate) using fsQCA 4.1. Their difference lies in the treatment of logical remainders: the complex solution uses none, the parsimonious solution uses all, and the intermediate solution uses only those consistent with directional expectations derived from theory and empirical evidence (20). For the intermediate solution, directional expectations are specified as follows: the presence of PC, AE, TC, and HC contributes to high governance effectiveness, while the absence of PR (i.e., lower PR) also contributes.

Following standard QCA practice, the intermediate solution serves as the primary basis for interpretation and the parsimonious solution to identify core and peripheral conditions. The intermediate solution yields three causal pathways and is, in this case, identical to the parsimonious solution.

Conditions appearing in both the intermediate and parsimonious solutions are treated as core conditions, while those appearing only in the intermediate solution are considered peripheral conditions. Because the two solutions coincide here, every condition entering the three pathways is a core condition and no peripheral conditions arise. Table 4 reports the three sufficient configurations through which African countries achieved high early-stage COVID-19 governance effectiveness, with each column representing a distinct pathway.

**Table 4:** Sufficient condition configuration analysis for achieving low early pandemic mortality.

Condition	Configuration A	Configuration B	Configuration C
PC	•		
AE		•	
TC			•
HC		⊗	⊗
PR	•		
Configuration Type	**Party-led high-risk response**	**Administrative compensation**	**Coercive compensation**
Consistency	0.942	0.896	0.859
Raw coverage	0.357	0.463	0.541
Unique coverage	0.049	0.052	0.112
Cases	Mozambique^*^, Uganda^*^, Côte d'Ivoire, Nigeria^*^	Senegal^*^, Ghana, Rwanda, Benin^*^, Tanzania, Ethiopia^*^, Gambia, Côte d'Ivoire	Malawi^*^, Gambia, Eritrea^*^, Tanzania, Ghana, Rwanda, Kenya^*^, Zimbabwe^*^
Solution consistency		0.851	
Solution coverage		0.652	

Source: Compiled by the author. Configurations A, B and C correspond to Pathways A, B and C in Section 3.5. •, core condition present; ⊗ = core condition absent; blank, condition may be either present or absent. Cases listed are those with membership above 0.5 in the configuration and above 0.5 in the outcome, ordered by configuration membership; cases marked with ^*^ are uniquely covered by that configuration. Three cases hold membership above 0.5 in a configuration but below 0.5 in the outcome and are deviant cases for consistency in kind: Burkina Faso and Niger (Configuration B) and Sierra Leone (Configuration C). Malawi is a deviant case for consistency in degree (0.952, 0.915).

In fuzzy-set QCA, the consistency of sufficiency configurations should generally exceed 0.75. The three configurational pathways identified in this paper all exceed this threshold comfortably, ranging from 0.859 to 0.942, and can be regarded as sufficient conditions for high early-stage pandemic governance effectiveness. The overall solution consistency is 0.851 and the solution coverage is 0.652, indicating that the three configurations jointly account for approximately 65 percent of the membership in the outcome set across the 52 African countries examined.

Ruling-party capacity appears in only one of the three pathways, but there it is a core condition in the pathway with the highest consistency (0.942). Its role is therefore conditional rather than general. Party capacity matters when transmission pressure is high, because organizational reach becomes critical to effective implementation. When transmission pressure is not high, party capacity does not distinguish successful from unsuccessful cases. The measure captures organizational reach, not the purposes for which that organization was built. What matters here is whether such reach can be mobilized when implementation becomes the binding constraint, not why it originally developed.

### 3.5 Interpretation of pathways

**Pathway A: party-led high-risk response (PC** ^*^ **PR)**

In Configuration A, PC and PR are core conditions, while AE, TC, and HC are not decisive. This pathway indicates that where transmission pressure is high, ruling-party organizational reach is sufficient for high early-stage governance effectiveness regardless of the level of state capacity. Consistency = 0.942, raw coverage = 0.357, and unique coverage = 0.049. This is the most consistent of the three pathways and also the narrowest in scope. Covered cases include Mozambique, Uganda, Côte d‘Ivoire, and Nigeria. All but Côte d'Ivoire are uniquely covered by this configuration.

In contexts of weak state capacity, ruling party infrastructural power functions as a substitutive informal institution (44, 45). Under high transmission risk, the ruling party's permanent local presence, routine organizational activity, and linkages to organized social constituencies translate into crisis governance capacity. Strong PC enhances prevention implementation through top-down policy transmission, grassroots information dissemination, and cross-level coordination, partially offsetting deficiencies in formal state capacity. The conjunction of the two core conditions is not incidental. Transmission risk is measured here in part by the share of the urban population living in informal settlements, and it is precisely in such settlements that formal administrative reach is thinnest. Where the epidemiological threat is concentrated in the parts of the population least accessible to the state, the organizational infrastructure of the ruling party becomes the channel through which containment measures can reach them at all.

Pathway B: administrative compensation (AE^*^ **~HC)**

In Configuration B, administrative AE and the absence of HC are the only core conditions. This pathway indicates that, under weak HC, high administrative execution is sufficient for high early-stage governance effectiveness. Consistency = 0.896, raw coverage = 0.463, and unique coverage = 0.052. Covered cases include Senegal, Ghana, Rwanda, Benin, Tanzania, Ethiopia, Gambia, and Côte d'Ivoire; Senegal, Benin and Ethiopia are uniquely covered by this configuration.

The absence of HC as a core condition does not imply that weak HC improves governance. Rather, in contexts where policy implementation capacity is strong and corruption is low, governance conditions may help mitigate the adverse effects of medical resource scarcity (46). This compensatory logic is central to understanding how resource-constrained states can nonetheless achieve effective pandemic governance. Governance capacity plays a decisive role in the effective allocation of limited public resources (47). Higher administrative execution ensures policy coherence from formulation to implementation, while lower corruption reduces resource leakage and distortion (48).

Pathway C: coercive compensation (TC^*^ **~HC)**

In Configuration C, TC and the absence of HC are the only core conditions. This pathway indicates that, under insufficient HC, strong coercive capacity can achieve high early-stage governance effectiveness. Consistency = 0.859, raw coverage = 0.541, and unique coverage = 0.112. Covered cases include Malawi, Gambia, Eritrea, Tanzania, Ghana, Rwanda, Kenya, and Zimbabwe; Malawi, Eritrea, Kenya and Zimbabwe are uniquely covered by this configuration.

Coercive capacity refers to the state's ability to maintain order, implement restrictive policies, and enforce pandemic measures. Stronger coercive capacity enables effective border controls, mobility restrictions, quarantines, and resource allocation. In many African countries, state reach varies substantially across national territory, particularly between administrative centers and more remote areas, which may affect the extent to which policies can be implemented uniformly (49, 50). Pathway C shares the absence of HC with Pathway B, meaning both represent compensatory governance pathways under insufficient HC. However, Pathway B relies on administrative AE, whereas Pathway C relies on state coercive capacity. The two pathways thus identify distinct compensating resources rather than variants of a single mechanism. Bureaucratic quality operates by allocating scarce resources coherently; territorial control operates by ensuring uniform enforcement across national territory.

The three pathways, while all sufficient for high governance effectiveness, embody distinct underlying logics of crisis response. Pathway A reflects a logic of mobilization substituting for execution: where formal state capacity is weak, the ruling party's organizational infrastructure bypasses the administrative system. Pathways B and C, by contrast, reflect a logic of specific capacity compensating for healthcare deficiency: administrative execution compensates through coherent resource allocation; coercive capacity compensates through territorial control. These are not variants of a single logic but qualitatively different mechanisms through which states overcome healthcare deficiencies. Pathway A relies on party-society linkages; Pathway B relies on bureaucratic quality; Pathway C relies on state coercive projection.

Synthesizing the three pathways reveals that African countries achieving high early-stage pandemic governance effectiveness exhibit both multiple conjunctural causation and equifinality.

First, PC is decisive in one configuration and does not enter the other two. It is a core condition where transmission pressure is high (Pathway A), the configuration with the highest consistency of the three, but does not enter the two compensatory pathways (Pathways B and C), which together account for the larger share of the outcome set. Party capacity is therefore not a general determinant of pandemic governance effectiveness in Africa but a resource whose relevance is conditional on the governance context. What the analysis establishes is the specific context in which it becomes decisive: where the pathogen circulates most readily among populations that formal administration reaches least well.

Second, the absence of HC is a core condition in Pathways B and C. This indicates that effective pandemic governance does not depend solely on the absolute level of public health systems, but on whether states can compensate for healthcare deficiencies through alternative resources, namely administrative execution or coercive capacity.

Finally, the three pathways represent distinct configurations of PC, state capacity, and governance context. Pathway A highlights the direct mobilizing role of PC under high transmission risk, while Pathways B and C reflect compensatory mechanisms that draw on state capacity alone amid public health constraints. These findings suggest that early-stage pandemic governance effectiveness is not driven by any single linear factor, but emerges from synergistic combinations of conditions within specific contexts. Collectively, the results affirm the explanatory value of the integrated “PC-state capacity-governance context” framework.

### 3.6 Robustness checks

Set-theoretic results depend on measurement decisions, case boundaries, and the calibration of analytical thresholds. Three families of checks are therefore reported. The first varies the measurement of party capacity, the study's focal condition. The second excludes the cases whose values were imputed rather than observed. The third varies the frequency, consistency, and PRI thresholds. All checks retain the parameters of the main model except where stated, and all are computed on the same calibrated data. Results are summarized in Table 5.

**Table 5:** Robustness checks.

Specification	Solution	Consistency	Coverage
Main model (*N* = 52)	PC^*^PR+ AE^*^~HC + TC^*^~HC	0.851	0.652
(a) Measurement of the focal condition			
PC = mean	PC^*^PR + PC^*^AE^*^~HC + PC^*^TC^*^~HC	0.837	0.651
PC = v2palocoff	PC^*^PR + PC^*^AE^*^~HC + PC^*^TC^*^~HC	0.845	0.636
PC = v2paactcom	PC^*^PR + PC^*^AE^*^~HC + PC^*^TC^*^~HC	0.853	0.652
PC = v2pasoctie	PC^*^~AE^*^PR + AE^*^~HC + TC^*^PR	0.872	0.552
(b) Scope of cases			
excl. PC-imputed cases (*N* = 49)	PC^*^PR + AE^*^~HC + TC^*^~HC	0.850	0.674
excl. Tourism-imputed cases (*N* = 49)	PC^*^PR + AE^*^~HC + TC^*^~HC	0.864	0.651
excl. both (*N* = 47)	PC^*^PR + AE^*^~HC + TC^*^~HC	0.863	0.670
(c) Analytical thresholds			
frequency ≥ 2	PC^*^TC^*^~HC^*^~PR + AE^*^~HC^*^~PR	0.851	0.531
raw consistency ≥ 0.85	PC^*^PR + AE^*^~HC + TC^*^PR	0.873	0.556
PRI ≥ 0.55	PC^*^~HC^*^PR + AE^*^~HC + TC^*^~HC +~AE^*^HC^*^PR	0.844	0.647

All specifications report the intermediate solution under the directional expectations specified above. In panel (a), the party-capacity condition is denoted PC throughout, whatever its specification in that row. Assigning the two cases whose v2svstterr value equals the sample median to either side of the crossover leaves the main solution unchanged. Panels (a), (b) and (c) correspond to the three families of robustness checks described in Section 3.6: measurement of the focal condition, scope of cases, and analytical thresholds. In the solution formulas, ^*^denotes logical AND (set intersection), + denotes logical OR (set union), and ~denotes the absence (negation) of a condition.

**(a) Measurement of the focal condition**

Because party capacity is conceptualized as a non-compensatory compound set, we test mean aggregation and each constituent indicator separately. Mean aggregation, local offices, and local activity preserve the broad three-pathway architecture but introduce PC into the two compensatory pathways. These specifications also expand the PC set, with 41–46 cases above 0.5 compared with 33 under the intersection rule, reducing discrimination and moving the condition closer to trivial necessity. Social ties behaves differently, retaining PC only in PC^*^~AE^*^PR. The intersection rule is therefore preferred because it defines a smaller, conceptually stricter set consistent with the non-compensatory conception of party capacity.

**(b) Scope of cases**

Two groups rely on 5th-percentile imputations: party capacity for Libya, Somalia, and Sudan, and importation risk for Equatorial Guinea, Liberia, and Somalia. Excluding either group (*N* = 49) or both (*N* = 47) reproduces the main three-pathway solution unchanged. Consistency ranges from 0.850 to 0.864 and coverage from 0.651 to 0.674. The findings therefore do not depend on imputed cases.

**(c) Analytical thresholds**

Lowering the PRI threshold to 0.55 adds one configuration, producing a fourth pathway with negligible unique coverage (0.018) and narrowing the party pathway to PC^*^~HC^*^PR, while the administrative and coercive pathways remain unchanged. Raising raw consistency to 0.85 replaces TC^*^~HC with TC^*^PR, indicating sensitivity in the exact specification of the coercive pathway, while TC itself remains stable. The frequency-threshold test produces the most substantial structural departure from the main solution. This is expected because seven of the eleven configurations coded 1 are single-case rows; a threshold of 2 therefore removes most of the information contained in the truth table.

## 4 Case analysis

Based on the sufficiency analysis results, this paper selects typical and deviant cases for in-depth analysis. The within-case analysis is used to assess the plausibility of mechanisms consistent with the configurational results, rather than to estimate the independent causal effect of any single condition. Typical cases are identified by three criteria: membership above 0.5 in a configuration and in the outcome, with the former not exceeding the latter; unique membership in that configuration, since cases covered by multiple configurations are less suitable for distinguishing between the mechanisms implied by individual configurations; and, among the cases satisfying both, the highest configuration membership (51). This yields Mozambique for Configuration A (0.748, 0.940), Senegal for Configuration B (0.839, 0.950), and Eritrea for Configuration C (0.860, 0.950).

### 4.1 Typical case for pathway A: Mozambique

Mozambique's pandemic vulnerability was shaped by three structural factors: fragile healthcare infrastructure, conflict-induced mass displacement, and a largely ungovernable urban periphery where an estimated 80% of the population resides beyond effective state reach (52). The latter reflects particularly low territorial control capacity (TC), the state's inability to project authority and enforce policies uniformly across its national territory. In formal QCA terms, Mozambique exhibited low TC and low HC, yet achieved high early-stage governance effectiveness through the mobilizing role of the ruling party's organizational capacity (PC). This places Mozambique squarely within Pathway A, where PC and high transmission risk (PR) are core conditions, while TC and HC are not decisive.

FRELIMO, the ruling party, has developed through decades of governance an organizational network deeply embedded within state institutions and extending into grassroots society (53). This network operates as a substitutive informal institution precisely where formal state capacity, especially territorial control, is weakest. The mechanism operates through three primary channels. First, regular inspection tours by central leaders enable top-down prevention decisions to bypass inefficient bureaucracy, ensuring rapid policy transmission even to remote areas where administrative presence is thin. Second, the party's capillary network reaches communities and villages, making party members key conduits of prevention information at the grassroots level. This trust-based communication has proven particularly effective in areas with low educational attainment and limited media access, precisely the populations that formal administration struggles to reach. Third, through internal coordination and ties to industry and commerce, the party network facilitates resource mobilization, enabling the government to introduce fiscal measures swiftly and guide shifts in enterprise production (54).

At the onset of the pandemic, cadres selected through FRELIMO's internal procedures promptly coordinated resources, disseminated policies, and reassured local populations, effectively mitigating the “policy attenuation” that typically arises from a weak administrative system (55). In this sense, the party's organizational infrastructure compensated for the state's limited territorial reach: where the state could not enforce, the party could persuade; where bureaucracy failed to deliver, the party network could transmit. These practices demonstrate that, under high transmission risk and weak territorial control, FRELIMO's organizational infrastructure served as a critical channel through which containment measures could reach vulnerable populations, exactly the substitutive logic captured by Pathway A.

### 4.2 Typical case for pathway B: Senegal

Senegal exemplifies the administrative compensation pathway (AE^*^~HC) identified by the QCA analysis. In 2020, Senegal possessed a mere 0.084 physicians per 1,000 inhabitants (56), far below the WHO benchmark of 2.28 doctors, nurses and midwives per 1,000 population, below which countries generally fail to achieve adequate coverage of essential health interventions (57). Healthcare infrastructure was also profoundly inequitable, with the capital Dakar harboring most high-quality facilities while peripheral and rural areas remained underserved.

Despite these severe healthcare deficiencies, Senegal achieved relatively low early-stage mortality and ranked second globally (after New Zealand) on Foreign Policy's COVID-19 Global Response Index (58). This success was made possible by strong administrative execution capacity, which compensated for medical scarcity through four mechanisms. First, efficient decision-making: President Macky Sall declared a state of emergency and imposed a curfew within days of the first confirmed case (59), drawing on a highly centralized administrative command system. Second, information transparency: the Ministry of Health established daily real-time reporting via television, radio, and online platforms, enhancing policy credibility (60). Third, rapid testing: the Institut Pasteur in Dakar, one of only two African laboratories capable of COVID-19 testing at the onset, delivered results within 24 h despite global supply shortages (61). Fourth, cross-sectoral coordination: mechanisms from previous disaster risk reduction frameworks were rapidly repurposed for pandemic response.

In QCA terms, Senegal's strong administrative execution paired with weak healthcare capacity precisely matches Pathway B, demonstrating that governance quality can mitigate medical resource scarcity where policy implementation is coherent and corruption is controlled.

### 4.3 Typical case for pathway C: Eritrea

Eritrea exemplifies the coercive compensation pathway (TC^*^~HC) identified by the QCA analysis. Like Senegal, Eritrea faced severe healthcare deficiencies and highly uneven distribution of medical resources. However, strong state coercive capacity successfully compensated for medical resource scarcity through four mechanisms: rapid lockdown, strict border control, mandatory quarantine, and zero-tolerance enforcement. As a result, Eritrea maintained extremely low case numbers during the early pandemic, ranking among the most effective countries globally (62).

After the first confirmed case was reported on 21 March 2020, the government declared a nationwide lockdown on 24 March, initially for 21 days, closing all schools, places of worship, and public spaces (63). This rapid decision-making derived directly from the state's highly centralized coercive enforcement system. Eritrea swiftly closed all land borders with Sudan and Ethiopia and suspended international flights. The government imposed a strict 21-day mandatory quarantine in centralized facilities for all incoming travelers, which was one of the most stringent measures in Africa (64). Security forces were deployed nationwide to enforce curfews and public gathering bans, with violators facing “the harshest legal sanctions” (65). The government utilized the existing local administrative network to ensure containment measures reached every community and village (62).

In QCA terms, Eritrea's strong coercive capacity paired with weak healthcare capacity precisely matches Pathway C, demonstrating that territorial control and uniform enforcement can compensate for medical deficiencies. Critically, however, this mechanism rests on a baseline of social trust in state authority, as illustrated by the contrasting case of Sierra Leone.

### 4.4 Deviant case for pathway C: Sierra Leone

In sufficiency analysis, deviant cases for consistency have higher membership in a configuration than in the outcome. They may be deviant in degree, with membership above 0.5 in both sets, or deviant in kind, with membership above 0.5 in the configuration but below 0.5 in the outcome (51). The latter poses the stronger challenge to sufficiency and is examined here. Three cases qualify as deviant cases for consistency in kind: Burkina Faso and Niger in Configuration B, and Sierra Leone in Configuration C. Burkina Faso (0.529) and Niger (0.503) have configuration memberships only marginally above the 0.5 crossover, making their classification more sensitive to calibration uncertainty and therefore less suitable for robust within-case inference. Sierra Leone, by contrast, has substantially higher configuration membership (0.707) and low outcome membership (0.182), making it the clearest deviant case for within-case analysis.

Sierra Leone serves as a deviant case for the coercive compensation pathway (TC^*^~HC). The country exhibited the core condition combination of Pathway C—strong coercive capacity (TC) paired with weak healthcare capacity (~HC)—yet failed to achieve high early-stage governance effectiveness.

Sierra Leone's coercive response was substantial. In March 2020, the government activated the National Public Health Emergency Operations Center, and in early April imposed a nationwide lockdown and curfew, with the military playing a significant role in enforcement (66).

However, containment outcomes were markedly worse than those of successful coercive-compensatory cases. By early May 2020, the country had recorded 155 confirmed cases and 8 deaths; by mid-June, cases had surged to 1,132 with 51 deaths (67). Relative to its population and health system capacity, epidemic control was notably ineffective.

The failure of coercive compensation in Sierra Leone stems from a fundamental precondition for the effective operation of coercive capacity: basic social trust in state authority, which is absent in the country. The protracted civil war left a deep legacy of institutional distrust (68); the Ebola epidemic (2014–2016) further exposed “persisting deep public suspicion and mistrust of the state” (69), and rumors of government corruption threatened mitigation efforts (70).

This historical distrust directly undermined coercive measures. Health personnel struggled to reach vulnerable populations in remote areas, social mobilization was weak, community engagement was limited, and security forces operated in a context where trust deficits remained unaddressed. Civil society organizations explicitly called for measures to “deepen citizens trust in the response process,” recognizing that trust is as important as other response measures (71).

The Sierra Leone case reveals a critical boundary condition for the coercive compensation pathway: coercive capacity can compensate for healthcare deficiencies only when it rests on a foundation of basic societal trust in state authority. Where such trust is absent, the state may possess enforcement tools but lacks the social legitimacy that renders coercive measures effective.

## 5 Conclusions and discussion

The QCA empirical analysis yields three principal findings, each of which directly engages with and qualifies one of the three theoretical perspectives reviewed in the literature: the state-centric, society-centric, and party-centric approaches.

First, ruling party capacity is a contextually contingent rather than universally critical resource. While the party-centric perspective highlights the pivotal role of political parties in Africa's state-building trajectory, our findings qualify this claim. PC appears as a core condition only in Pathway A, the most consistent but narrowest configuration. It does not enter the two compensatory pathways, which together cover a larger share of cases. Party capacity becomes decisive only where transmission pressure is high and formal administrative reach is thinnest, among populations least accessible to the state. Its relevance is therefore conditional on specific governance contexts, supporting the view that party capacity should be understood as a crisis-contingent resource rather than a generalized governance asset.

Second, state capacity dimensions perform differentiated compensatory functions. The state-centric approach emphasizes the multidimensionality of state capacity, and our findings specify how distinct dimensions operate under healthcare deficiency. The absence of healthcare capacity (~HC) is a core condition in both Pathway B and Pathway C, indicating that states can compensate for medical deficiencies through alternative resources. Administrative execution compensates through coherent resource allocation and low-corruption implementation, as illustrated by Senegal's centralized command system, information transparency, and cross-sectoral coordination. Coercive capacity compensates through territorial control and uniform enforcement, as demonstrated by Eritrea's border closures, mandatory quarantine, and zero-tolerance compliance. Importantly, coercive capacity is not self-sufficient: its conversion into compliance presupposes a baseline of societal trust in state authority. Sierra Leone, the deviant case for Pathway C, exhibited the core condition combination of that pathway—strong territorial control paired with weak healthcare capacity—yet failed to contain the epidemic, precisely because the legacy of civil war and of the Ebola response had left that trust absent. These findings move beyond the state-centric perspective by revealing not only that state capacity matters, but which dimensions matter, how they compensate for one another, and under what conditions they fail.

Third, the results affirm equifinality and conjunctural causation. High governance effectiveness emerges through three distinct pathways, with no single factor universally decisive. This finding resonates with the society-centric perspective's emphasis on contextual variation and the importance of local actors, while simultaneously transcending its tendency to idealize civil society or rely on reductive “neo-patrimonialism” frameworks. The configurational approach shifts analytical focus from the conventional question of “which factor matters most” to a more nuanced inquiry: “which combinations of conditions work best, under what specific circumstances, and through what mechanisms?”

This study makes two primary theoretical contributions.

First, the study reconceptualizes party capacity as a context-dependent governance resource rather than a universally applicable governance asset. By demonstrating that PC becomes decisive only under the specific conjunction of high transmission risk and thin administrative reach, the study qualifies the party-centric perspective and integrates it into a broader configurational framework.

Second, the study disaggregates the multidimensionality of state capacity and reveals the differentiated compensatory functions of its distinct dimensions in crisis settings. Administrative execution compensates through coherent resource allocation and low-corruption implementation; coercive capacity compensates through territorial control and uniform enforcement.

The scope conditions of this study confine its findings to the African sample, the early pandemic period (March to May 2020), and the limitations of reported mortality data. The configurational associations identified should be interpreted as patterns in the data rather than causal claims, and the conversion of party capacity into governance effectiveness is understood as a crisis-specific mechanism, not a model of routine governance. The within-case analyses of Mozambique, Senegal, Eritrea, and Sierra Leone are intended to illustrate the plausibility of the mechanisms implied by each pathway, not to provide definitive causal tests.

One limitation concerns the use of reported deaths as the outcome. AE, TC, and HC may affect death ascertainment and reporting, introducing systematic measurement error correlated with the conditions and potentially altering the configurations identified. To the extent that stronger AE and TC improve registration, they would increase reported mortality and lower membership in the outcome set. This reporting effect would bias against the association of AE and TC with low reported mortality in Pathways B and C, respectively. Low HC, measured as physicians per 10,000, may instead reduce the diagnosis and certification of COVID-19 deaths, increasing outcome membership in the same direction as the observed association. Because the absence of HC is a core condition in both pathways, differential certification offers an alternative explanation for the compensatory findings. The insufficiency of weak HC alone does not rule out this possibility. The present analysis cannot establish the combined impact of these reporting processes or exclude their influence on the configurational structure. The interpretation of Pathways B and C as compensatory mechanisms therefore remains provisional pending validation with independent mortality evidence.

Future research could proceed in the following directions. First, temporal QCA methods could be employed to track the dynamic evolution of pandemic governance over time. Second, later stages of the pandemic or other public health crises in Africa could be examined to validate and extend the framework. Third, process-tracing methods could be integrated to conduct deeper causal mechanism tests in selected cases, with particular attention to the operational processes of ruling-party grassroots organizations in pandemic prevention and control. Such mixed-method designs would help move beyond the configurational associations identified here toward a more robust understanding of causal processes (72). Fourth, future research could incorporate community-level indicators to better capture the grassroots mobilization mechanisms that macro-level variables cannot adequately represent.

## Data Availability

Publicly available datasets were analyzed in this study. The data sources are cited in Sections 3.2 and 3.3. The raw data and calibrated set-membership scores for all 52 cases are provided in the “Raw Data” and “Processed Data” worksheets, respectively, of Supplementary Table 1. Further inquiries can be directed to the corresponding author.

## Footnotes

1 The ruling party is identified as follows. First, it is the party of the person actually serving as head of government between 1 March and 31 May 2020; in presidential systems where the president also serves as head of government, or where no separate premiership exists, the president's party is used. Under coalition government, the specific party of the head of government is taken as the unit of identification, rather than the governing coalition as a whole. Second, where the head of government was non-partisan, the party holding the largest number of seats in the national legislature during the same period is used. Third, where none of these criteria yields a party of national political significance—because no national legislature was functioning effectively, because parliamentary representation was not organized along party lines, or because state authority was territorially divided among competing governments—no ruling party is assigned, and the case is coded as having no identifiable national ruling party. On this basis, 46 countries are assigned observed V-Party values for their ruling party; three (Central African Republic, Ethiopia, The Gambia) are assigned the closest feasible proxy observation; and three (Libya, Somalia, Sudan) are coded as having no identifiable national ruling party. Because party capacity is defined as the national organizational reach of the ruling party, the absence of any party exercising that role is treated as a substantively low value rather than as missing data, and these cases are assigned the 5th percentile of the sample distribution.

2 The V-Party indicators are latent-variable estimates generated through a Bayesian item response theory measurement model. The resulting scores are expressed on a standardized interval scale similar to a Z-score, with 0 approximately representing the mean across all country-party-years in the V-Party data and values typically falling between −5 and +5. Accordingly, this study uses 0 as the crossover point, and takes −1 and +1 as the theoretical anchors for full non-membership and full membership, respectively.

3 GE_2019 and CC_2019 are zero-centered standardized indicators, but their distribution across the African sample in this study is notably skewed toward negative values. Applying the theoretical anchors of−1, 0, and +1 directly would result in a large number of cases clustering in the low membership range, undermining calibration discrimination. Accordingly, this study adopts the 5th, 50th, and 95th percentiles of the actual sample distribution as anchors for direct calibration.

4 The three anchors are 64.04, 91.58 and 99.67. Two cases take a value on v2svstterr identical to the sample median (91.57), and would therefore receive a membership score of exactly 0.50 if the median were used as the crossover—a value that can be assigned to neither the set nor its complement once fuzzy scores are dichotomized for truth-table construction (20, 22). The crossover is accordingly placed marginally above the median. The adjustment alters no membership score by more than 0.001 and leaves the crisp coding of every case on TC unchanged.

5 The original distribution of the number of physicians per 10,000 population is markedly right-skewed (skewness = 2.43). Direct calibration would render the measure susceptible to distortion by a few high-value cases, thereby diminishing sample discrimination. To address the long-tailed distribution and reduce the impact of extreme values, this study applies a logarithmic transformation to the variable prior to conducting fuzzy-set calibration. Because reporting years vary across countries, values are drawn from the most recent year available up to and including 2019.

6 Because reporting years vary across countries, values are drawn from the most recent year available up to and including 2019. Where no valid value exists (Equatorial Guinea, Liberia, Somalia), the 5th percentile of the sample distribution is imputed; this reflects the structurally low, rather than absolute zero, exposure characteristic of these countries, while avoiding the distributional distortions associated with case deletion, mean imputation, or direct zero assignment. Given the pronounced right-skewness of the raw distribution, a logarithmic transformation is additionally applied to mitigate the impact of extreme values and improve calibration discrimination.

7 The PRI threshold is set at 0.65 because the distribution of PRI values contains a pronounced gap at this point: the eleventh-ranked configuration records a PRI of 0.657 and the twelfth a PRI of 0.584, a difference of 0.073 that is the largest anywhere in the distribution. Locating the threshold within an empirical gap rather than at a conventional value avoids separating configurations of substantively similar quality.
